# Interpretation of personal genome sequencing data in terms of disease ranks based on mutual information

**DOI:** 10.1186/1755-8794-8-S2-S4

**Published:** 2015-05-29

**Authors:** Young-Ji Na, Kyung-Ah Sohn, Ju Han Kim

**Affiliations:** 1 Department of Biology, University of Pennsylvania, Philadelphia, PA 19104, USA; 2 Department of Information and Computer Engineering, College of Information Technology, Ajou University, Suwon 443-749, Korea; 3 Systems Biomedical Informatics Research Center, College of Medicine, Seoul National University, Seoul 110-799, Korea; 4 Seoul National University Biomedical Informatics, Division of Biomedical Informatics, College of Medicine, Seoul National University, Seoul 110-799, Korea

**Keywords:** Next-generation sequencing, MeSH tree structure, disease risk, personal genome

## Abstract

**Background:**

The rapid advances in genome sequencing technologies have resulted in an unprecedented number of genome variations being discovered in humans. However, there has been very limited coverage of interpretation of the personal genome sequencing data in terms of diseases.

**Methods:**

In this paper we present the first computational analysis scheme for interpreting personal genome data by simultaneously considering the functional impact of damaging variants and curated disease-gene association data. This method is based on mutual information as a measure of the relative closeness between the personal genome and diseases. We hypothesize that a higher mutual information score implies that the personal genome is more susceptible to a particular disease than other diseases.

**Results:**

The method was applied to the sequencing data of 50 acute myeloid leukemia (AML) patients in The Cancer Genome Atlas. The utility of associations between a disease and the personal genome was explored using data of healthy (control) people obtained from the 1000 Genomes Project. The ranks of the disease terms in the AML patient group were compared with those in the healthy control group using "Leukemia, Myeloid, Acute" (C04.557.337.539.550) as the corresponding MeSH disease term.

The mutual information rank of the disease term was substantially higher in the AML patient group than in the healthy control group, which demonstrates that the proposed methodology can be successfully applied to infer associations between the personal genome and diseases.

**Conclusions:**

Overall, the area under the receiver operating characteristics curve was significantly larger for the AML patient data than for the healthy controls. This methodology could contribute to consequential discoveries and explanations for mining personal genome sequencing data in terms of diseases, and have versatility with respect to genomic-based knowledge such as drug-gene and environmental-factor-gene interactions.

## Background

The advent of next-generation sequencing (NGS) technologies has had a huge impact on functional genomics [1]. NGS technologies have already been employed to sequence the constitutional genomes of several individuals [2-7]. The first five cancer genomes to be found contained thousands of novel somatic mutations and implicated new genes in tumor development and progression [8-13]. Current knowledge of the genetic variants that underlie disease susceptibility, treatment response, and other phenotypes will continually improve as these types of investigation expand the catalog of DNA sequence variation in humans. However, a far greater challenge is mining genomes for clinically useful information. Present analytical methods are insufficient to make genetic data accessible in a clinical context, and the clinical usefulness of these data for individual patients has not been formally assessed.

Despite the existence of more comprehensive databases and better methods for analyzing genetic variants, genome interpretation remains an elusive goal. Ashley and colleagues [14] made an impressive and ambitious effort to use full-genome sequence data in the clinical setting. They estimated a patient's risk of several common diseases using several types of information, including single-nucleotide polymorphisms that have been associated with the risk of these diseases. In addition, Chen and colleagues [15] tracked down genetic effects in the genotype-phenotype chain to discover relevant biomarkers for further personalization of diagnoses and therapeutics. Data from studies of disease concordance in monozygotic twins suggest that a negative test result from whole-genome sequencing data for many common diseases (e.g., cancer) would not appreciably reduce an individual's risk relative to that of the baseline population [16]. Focusing computational analysis schemes on personal genomes and diseases has the potential to bridge this knowledge gap.

The aim of the present study was to associate the personal genome with disease predisposition patterns based on calculations of the closeness between the genome and diseases. This research provides the first method for associating the personal genome with diseases with regard to genome-sequencing studies. The method works by ranking all variants in the personal genome as potential disease risks, and reporting Medical Subject Headings (MeSH) terms that are significantly associated with highly ranked genes. A key distinguishing feature of this methodology is that the algorithm simultaneously considers the functional impact of damaging variants and curated disease-gene associations. This research is especially important when pathologic variants exhibit characteristic trends or properties specific to a given disease.

## Methods

**Figure 1:**
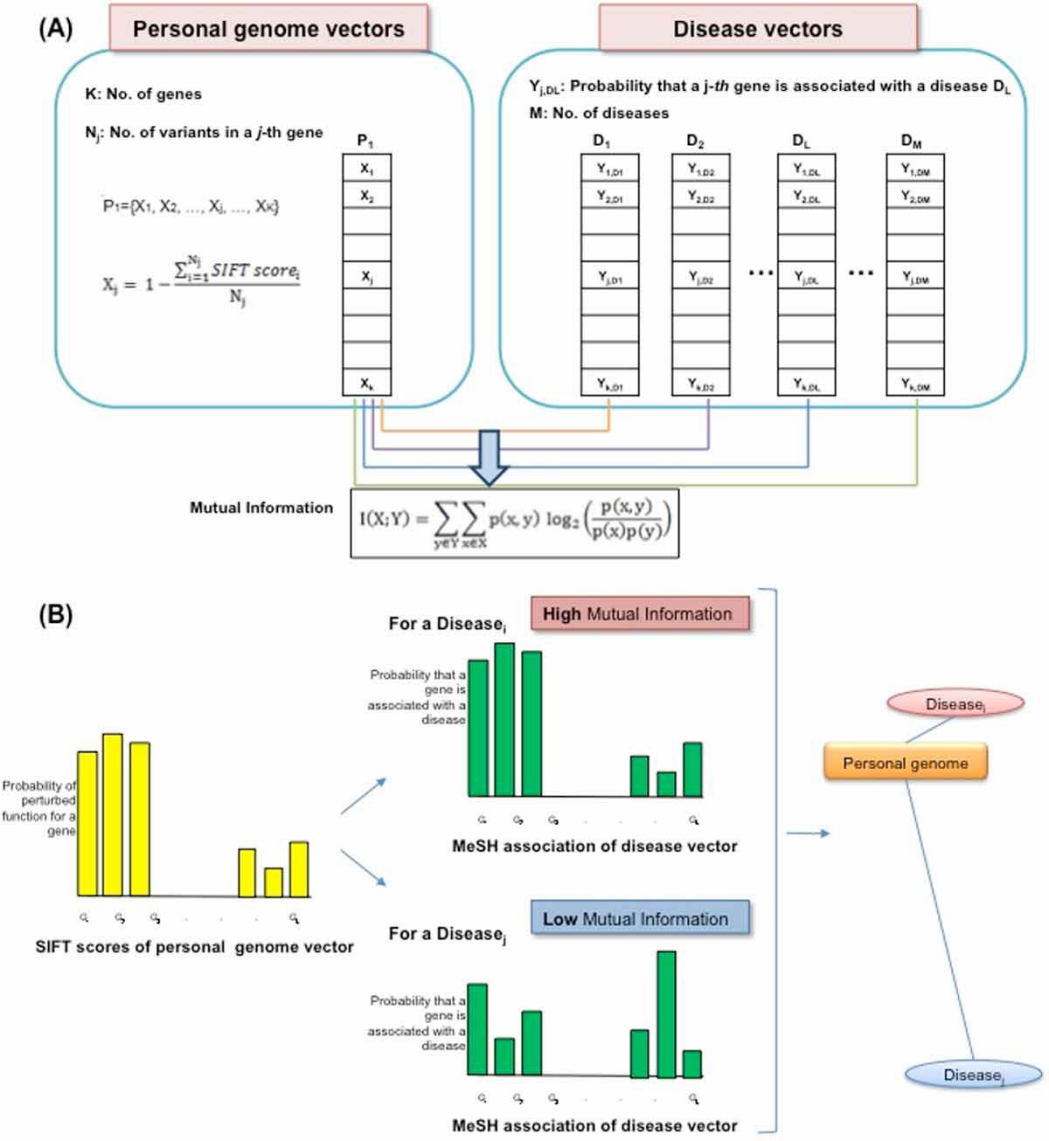
**Overview of the methodology**. This analysis scheme is based on mutual information as a measure of the relative closeness between the personal genome and diseases. (A) Calculating mutual information between the personal genome and diseases. The closeness between the personal genome and diseases was measured by considering both damaging missense mutations and knowledge of disease-gene associations. (B) Discovering association patterns based on mutual information as a measure of the relative closeness between the personal genome and diseases. The yellow-bars plot indicates VSiftVar and the green-bars plot indicates VDisGene.

The aim of the method described herein is to measure the closeness between a personal genome and a disease. We considered both damaging missense mutations and knowledge of disease-gene associations. The overall procedure for computing the closeness is as follows (Figure 1A):

• Step 1. Quantify damaging missense mutations in personal genome sequences using the Sorting Intolerant From Tolerant (SIFT) tool [17].

• Step 2. Extract knowledge about disease-gene associations from Online Mendelian Inheritance in Man (OMIM) by simultaneously considering the hierarchical structure of MeSH.

• Step 3. Pairwise computation of mutual information between the SIFT score vectors of variants in the personal genomes and the disease-gene association vectors.

• Step 4. Analyze the similarity structure pattern between personal genomes and diseases.

### Personal genome sequencing data

We obtained targeted exome sequencing data from The Cancer Genome Atlas (TCGA). The platform of mRNA expression was Illumina HiSeq 2000 RNA Sequencing V2. Illumina 2 × 100-bp paired-end sequencing reads were produced after elution from capture arrays. Illumina paired-end reads were aligned to NCBI build36 using BWA 0.5.5. Somatic mutations were identified using SomaticSniper and a modified version of the SAMtools indel caller. The TCGA data set has 494 variants identified in 50 patients with acute myeloid leukemia (AML) by targeted resequencing of 601 genes having 7,932 coding exons. To show comparisons of other diseases groups with healthy controls in the 1000 Genomes Project, we obtained high-throughput sequencing data in several cancer types such as bladder cancer, breast cancer, colon cancer, kidney cancer, lung adenocarcinoma, lung squamous cell carcinoma, malignant melanoma, ovarian serous cystadenocarcinoma, prostate cancer and rectal cancer from TCGA and breast cancer and lung cancer from Catalogue Of Somatic Mutations In Cancer (COSMIC). The 1000 Genomes Project provides 1,092 whole-genome sequences obtained from 14 populations drawn from Europe, East Asia, sub-Saharan Africa, and the Americas [18].

### Functional impact of nonsynonymous variants

In order to measure the relative closeness between personal genomes and diseases, we created SIFT score vectors of variants (VSiftVar) for the personal genome sequences, and binary vectors for disease-gene association (VDisGene) obtained from OMIM. To assess the effect of a substitution, SIFT assumes that important positions in a protein sequence have been conserved throughout evolution, and substitutions at these positions may affect protein function. By using sequence homology, SIFT predicts the effects of all possible substitutions at each position in the protein sequence. VSiftVar was created for each personal genome sequence. Let *X_j _*denote the probability of a perturbed or altered function of a gene, *G_j_*. The original SIFT scores range from 0 to 1. If a SIFT score is smaller than 0.05, it is predicted to be "damaging"; otherwise, it is predicted to be "tolerated." *X_j _*is defined as 1 minus the average SIFT scores for all nonsynonymous variants i of *G_j_*, denoted as *G_ji_*. The newly defined score still ranges from 0 to 1, and a larger score means the variant is more likely to be deleterious:

$$Xj=1-\frac{\overset{\left|Gji\right|}{\underset{i=1}{\sum }}SIFTscorei}{\left|Gji\right|}$$

The length of VSiftVar equals the total number of genes in OMIM.

### Disease-gene association based on the MeSH tree structure

We obtained a list of disorders, disease genes, and associations between them from OMIM, which listed 5,911 disorders and 2,721 disease genes as of November 2011. We applied the MeSH controlled vocabulary to the disease features from OMIM to organize diseases for the following analyses.

We attempted to use the MeSH controlled vocabulary to organize the disease features referred to in OMIM. The extracted disease names from OMIM were mapped to the MeSH terms in two successive term-matching steps. First, we looked for exact matches, where all words composing the name had an identical corresponding MeSH term, and vice versa (the word order and the case were not considered). When this step failed, we looked for partial matches by at least two words.

The MeSH tree contains a finite set of MeSH codes, *M*. A specific variant, *v *∈ *V*, is associated with zero, one, or more MeSH codes [i.e., forms a set M_v_={m: annot(*v,m*) ∩ m ∈ M}, where the predicate annot() pairs variants with their MeSH codes]. VDisGene is a binary vector indicating either disease association or no disease association for each gene in the MeSH tree. Let *Y_j _*denote the binary disease-association value of a gene *j *(e.g., *Y_j _*= 0 for no disease association and *Y_j _*= 1 for disease association for the corresponding gene, *j*). The length of the disease vector is also the total number of genes in OMIM.

### Measuring relative closeness between the personal genome and diseases

We applied mutual information as the measure of the relative closeness between the personal genome and the diseases. Formally, the mutual information of VSiftVar, denoted as *X*, and VDisGene, denoted as *Y*, can be defined as

$$I\left(X;Y\right)=\underset{y\in Y}{\sum }\underset{x\in X}{\sum }p\left(x,y\right)log2\left(\frac{p\left(x,y\right)}{p\left(x\right)p\left(y\right)}\right)$$

where *p(x, y) *is the joint probability distribution function of *X *and *Y, p(x) *is the marginal probability distribution function of *X*, and *p(y) *is the marginal probability function of *Y*.

Higher mutual information between VSiftVar of a personal genome and VDisGene of a disease from OMIM means that the personal genome is more likely to be associated with the disease. Zero mutual information means that the joint distribution of the personal genome-disease association contains no more information than the personal genome-disease relationship considered separately (Figure 1B). This means that mutual information can be used as a metric between SIFT scores of variants in the personal genome sequencing data and the disease-gene association obtained from OMIM related to their degree of independence. We hypothesized that a higher mutual information score between VSiftVar of a personal genome and VDisGene of a disease implies that the personal genome is more susceptible to the disease than other diseases.

### Characterization of 1000 Genomes Project data according to the population

In order to identify the similarity structure pattern between personal genomes and diseases according to subpopulations, we clustered individuals in the data set based on mutual information between VSiftVar and VDisGene using a hierarchical clustering method (distance measure, Manhattan; linkage method, Median).

To compare the variability of mutual information with regard to MeSH disease categories in the entire population, we performed analysis of variance (ANOVA) to test whether each MeSH disease category exhibited a statistically significant different relative closeness with the personal genomes according to the population. Differences among each of the subpopulations was then tested using a post-ANOVA Tukey's HSD test for post-ANOVA comparisons, because a significant F-ratio shows only that the aggregate difference among the means of several samples is significantly greater than zero.

## Results

### Functional impact of nonsynonymous variants of AML patients and healthy controls in the 1000 Genomes Project

To identify the different extent of damaging effects of variants between patients and healthy controls, we obtained SIFT scores for 494 variants in 447 genes from 50 unrelated AML patients, and extracted SIFT scores for the variants of the same genes in the healthy controls from the 1000 Genomes Project data. We obtained 1,935, 1,713, and 2,312 variants from Europeans, Asians, and Africans, respectively. The distribution of SIFT scores was categorized into seven groups (Table 1). SIFT assigns a "functional importance" score to variants with a default cutoff threshold of 0.05, with variants with a SIFT score higher than this threshold regarded as "benign." Among the AML patients, 41.30% of the variants exhibit SIFT scores of < 0.05 and were thus designated as variants that are functionally damaging. The proportions of damaging variants among the healthy controls were 32.50%, 34.62%, and 34.43% for Europeans, Asians, and Africans, respectively. In order to show that AML patients had statistically more variants with a damaging impact, we performed Fisher's exact test using the number of variants with damaging (< 0.05) and non-damaging (>= 0.05) effect because a SIFT score is smaller than 0.05, it is predicted to be "damaging"; otherwise, it is predicted to be "tolerated." The Fisher's exact test showed that AML patients had statistically more variants with a damaging impact than healthy controls in the 1000 Genome Project data (*P *= 6.441e-08, African controls; *P *= 1.808e-08, European controls; *P *= 1.206e-06, Asian controls).

**Table 1:** Distribution of variants by SIFT score in the AML patients and the healthy controls

SIFT		AML patients		Healthy controls in the 1000 Genomes Project					
**Score**	**Impact**	**No. of variants**	**%**	**No. of variants**	**%**	**No. of variants**	**%**	**No. of variants**	**%**

0	Damaging	131	26.52	421	18.21	339	17.51	333	19.44
0.001-0.050	Damaging	73	14.78	375	16.22	290	14.99	260	15.18
0.051-0.100	Potentially damaging	25	5.06	173	7.48	146	7.55	129	7.53
0.101-0.200	Borderline	28	5.67	295	12.76	242	12.51	190	11.09
0.201-0.500	Tolerant	63	12.75	441	19.07	358	18.50	326	19.03
0.501-0.999	Tolerant	53	10.73	298	12.89	245	12.66	212	12.38
1.00	Tolerant	121	24.49	309	13.37	315	16.28	263	15.35
Total		494	100.00	2312	100.00	1935	100.00	1713	100.00

### Similarity structure pattern of personal genomes in the 1000 Genomes Project and diseases

**Figure 2:**
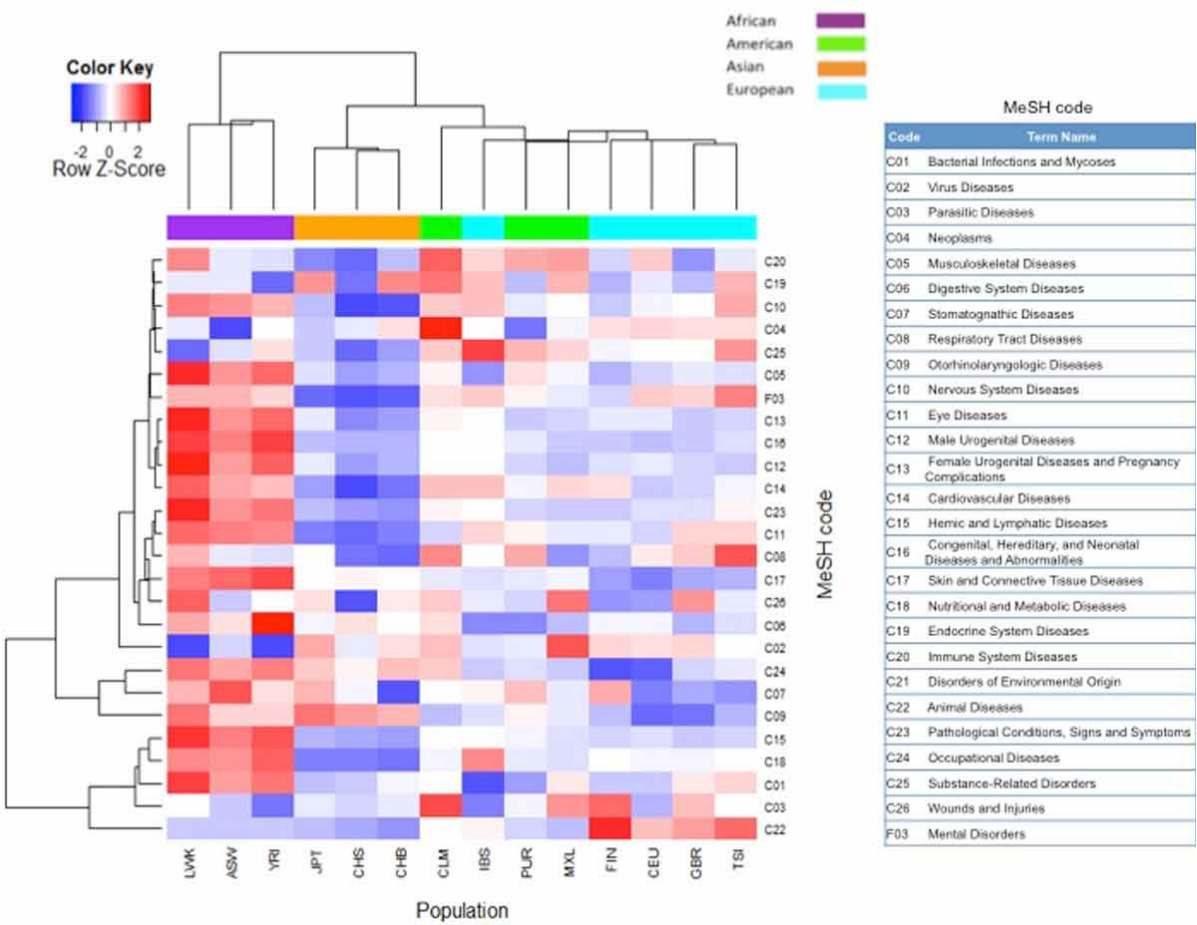
**Heatmap of mutual information of diseases according to the population in the 1000 Genomes Project**. The heatmap shows mutual information of diseases in the MeSH tree according to data from the population in the 1000 Genomes Project. Column colors: purple, African; orange, Asian; green, American; cyan, European.

It is necessary to understand overall relative patterns of disease association in healthy people, because the 1000 Genomes Project data are important as reference genome sequencing data. A hierarchical clustering method using mutual information between VSiftVar and VDisGene was used to detect relative closeness patterns of subpopulations in the 1000 Genomes Project data and diseases. Data from subjects belonging to African subpopulations - comprising Yoruba in Ibadan, Nigeria (YRI), Luhya in Webuye, Kenya (LWK), and those with African ancestry in southwest USA (ASW) - were clustered into a single group. The overall mutual information values are higher in these African subpopulations than in other subpopulations (Figure 2).

The differences in relative closeness between subpopulation genomes and diseases with regard to MeSH disease categories were explored by performing ANOVA using mutual information between VSiftVar and VDisGene with the entire 1000 Genomes Project data (Table 2). We found that three MeSH categories exhibited statistically significantly different relative closeness among subpopulations in the 1000 Genomes Project data: C15 - hemic and lymphatic diseases (*P *= 2.58E-07); C16 - congenital, hereditary, and neonatal diseases and abnormalities (*P *= 1.19E-06); and C17 - skin and connective-tissue diseases (*P *= 1.19E-06). These MeSH disease categories have higher mutual information in African populations than in any other subpopulations (Additional file 1). Moreover, a post-ANOVA Tukey's HSD test showed that these MeSH disease categories have significantly different mutual information in African subpopulations (Additional file 1).

**Table 2:** Statistically significant differences of MeSH codes among the healthy controls

Code	Term	*P *(ANOVA)
C01	Bacterial Infections and Mycoses	0.01400
C02	Virus Diseases	0.01435
C03	Parasitic Diseases	0.22684
C04	Neoplasms	0.55150
C05	Musculoskeletal Diseases	0.00003
C06	Digestive System Diseases	0.02709
C07	Stomatognathic Diseases	0.20351
C08	Respiratory Tract Diseases	0.23714
C09	Otorhinolaryngologic Diseases	0.00055
C10	Nervous System Diseases	0.00139
C11	Eye Diseases	0.00002
C12	Male Urogenital Diseases	0.00013
C13	Female Urogenital Diseases and Pregnancy Complications	0.00013
C14	Cardiovascular Diseases	0.00058
C15	Hemic and Lymphatic Diseases	**2.58E-07**
C16	Congenital, Hereditary, and Neonatal Diseases and Abnormalities	**1.19E-06**
C17	Skin and Connective-Tissue Diseases	**1.19E-06**
C18	Nutritional and Metabolic Diseases	0.00009
C19	Endocrine System Diseases	0.51232
C20	Immune System Diseases	0.00852
C22	Animal Diseases	0.00130
C23	Pathological Conditions, Signs, and Symptoms	0.00005
C24	Occupational Diseases	0.00149
C25	Substance-Related Disorders	0.04237
C26	Wounds and Injuries	0.59611
F03	Mental Disorders	0.00019

**Figure 3:**
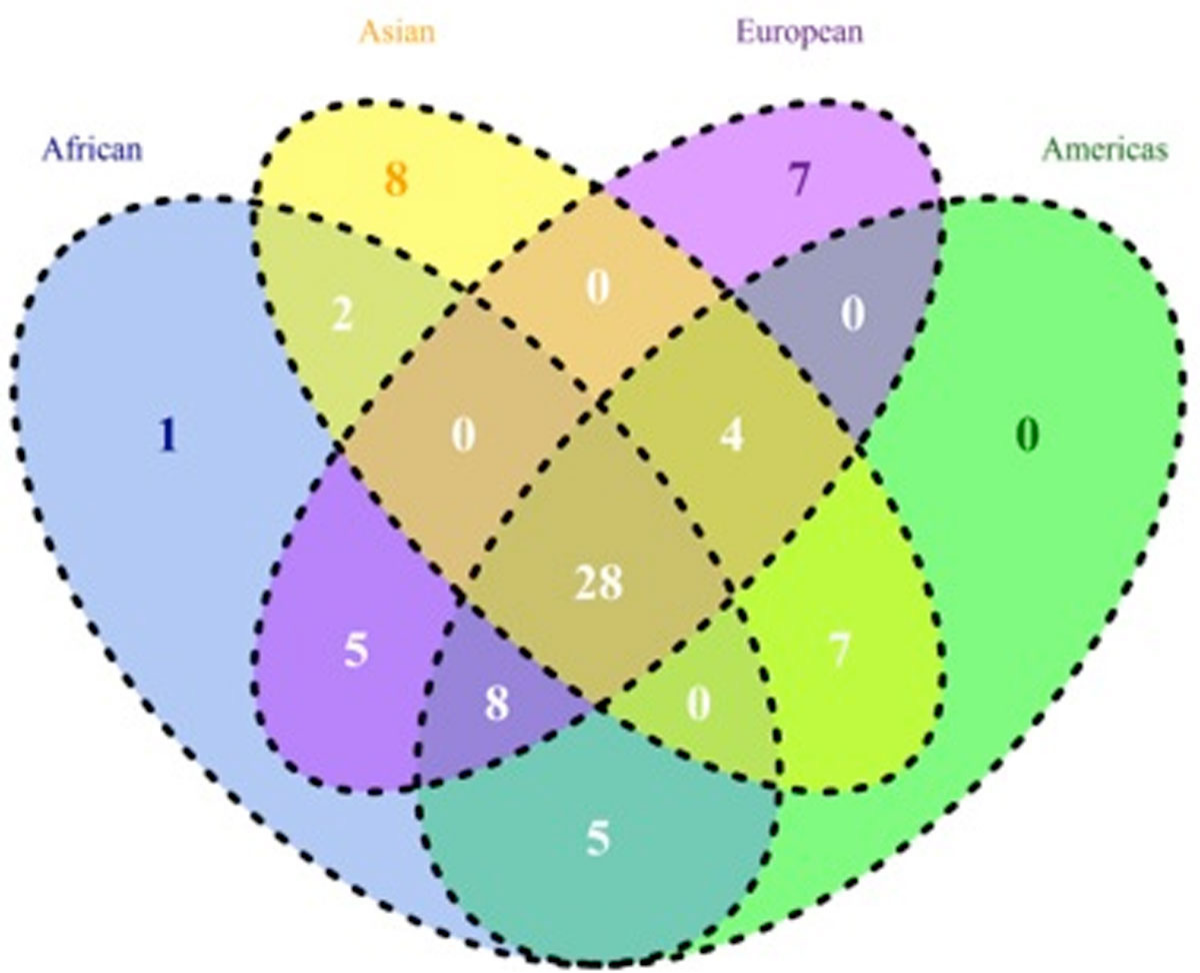
**Venn diagram of the top-50 diseases in the 1000 Genome Project**. Circle colors in the Venn diagram indicate African as blue, Asian as yellow, American as green, and European as violet. The numbers shown in this diagram represent the number of diseases in each intersection area or set.

The top-ranked diseases in common among subpopulations were determined showing a Venn diagram of the top-50 diseases from subpopulations in the 1000 Genomes Project data (Figure 3). About 56% of the top-50 diseases (28/50) are common in subpopulations; in contrast, macular degeneration (C11.768.585.439) is only associated with African subjects. In particular, arthritis (C05.550.114), ichthyosis vulgaris (C16.131.831.512.410, C16.320.850.405, C17.800.428.333.410, C17.800.804.512.410, and C17.800.827.405) and viremia (C02.937, C23.550.470.790.500.900) are only associated with Asian subpopulations, and AIDS-related complex (C02.782.815.616.400.080, C02.800.801.400.080, C02.839.080, and C20.673.480.080) and hematuria (C12.777.934.442, C13.351.968.934.442, and C23.550.414.849) are only associated with European subpopulations.

### Relative disease-rank patterns between patients and healthy subpopulations in the 1000 Genomes Project

In the genome sequencing data from a patient with a specific disease, if the rank of the corresponding disease term based on mutual information is relatively high, this could support that this proposed methodology is appropriate for associating the personal genome with the disease. Before comparing ranks of disease terms in the patient sequencing data, we ranked disease terms in healthy people in the 1000 Genome Project data as control data under all of the categories "disease" of the MeSH (Additional file 2). As expected, the distribution of disease terms in healthy people was ranked randomly overall; in other words, we cannot link a healthy person's genome to any particular disease.

**Figure 4:**
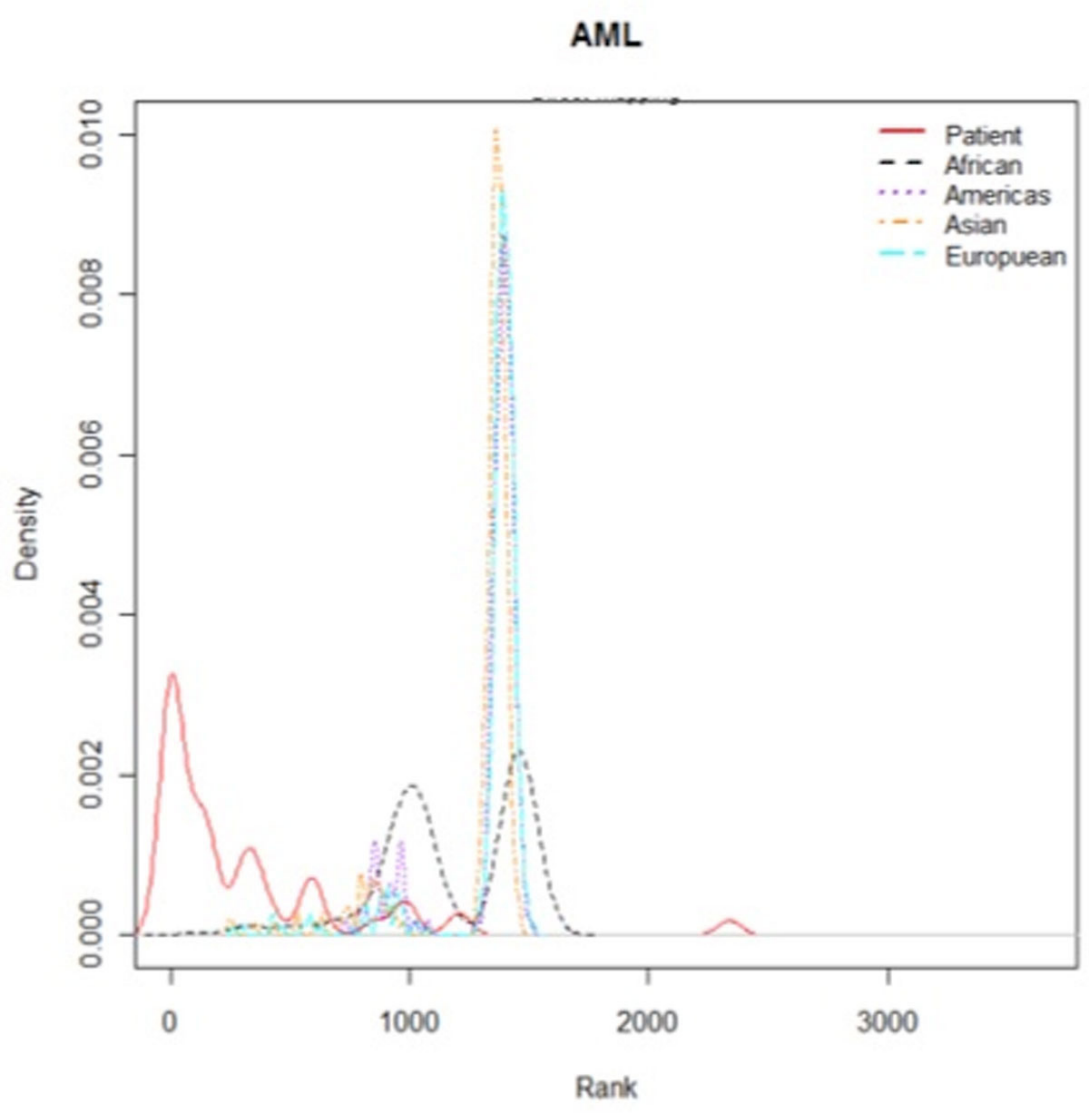
**Distribution of the rank based on mutual information**. The density plot shows the rank based on mutual information of the MeSH disease term "leukemia, myeloid, acute" (C04.557.337.539.550) between AML patients and healthy controls in the 1000 Genomes Project.

**Figure 5:**
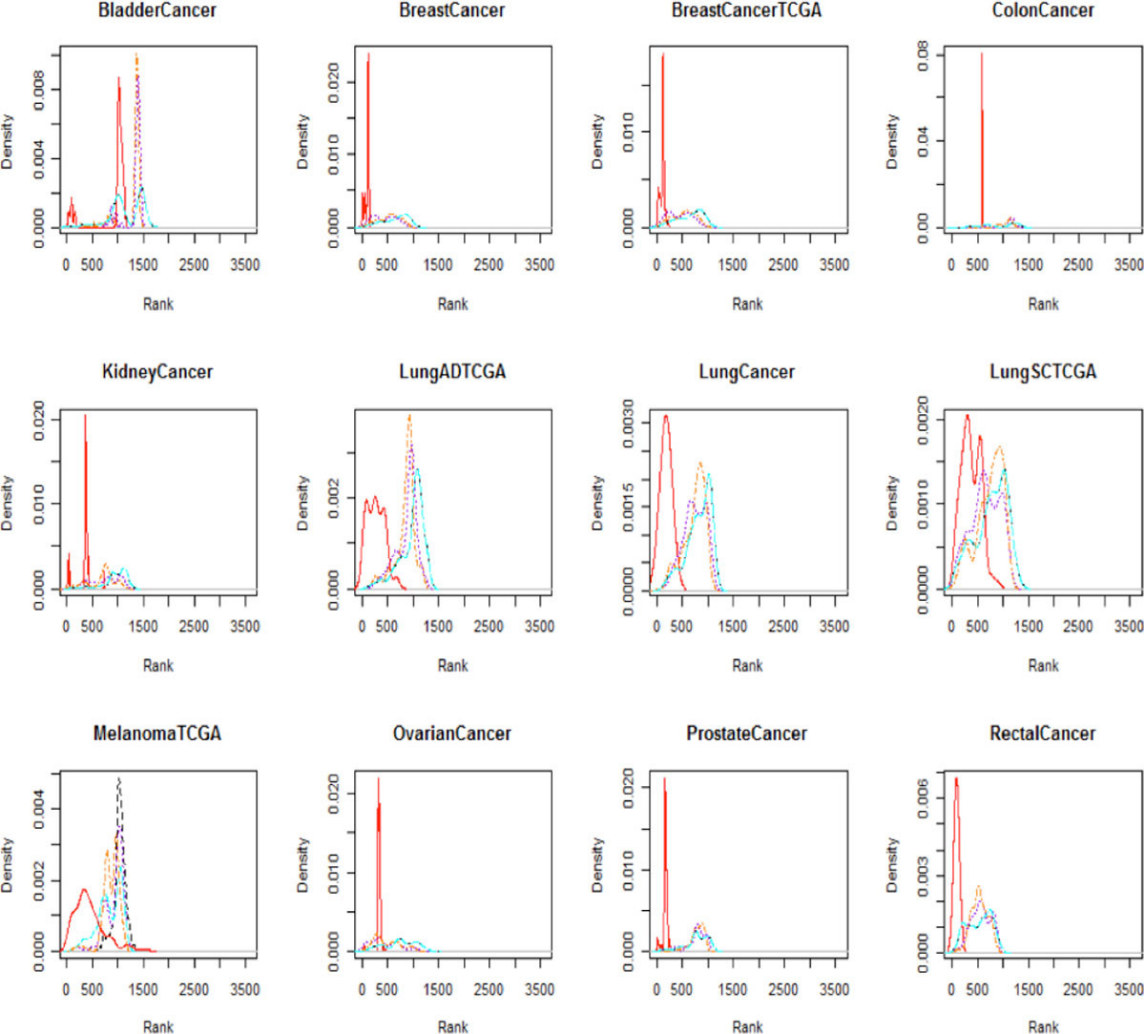
**The rank of the MeSH disease term between several disease groups and healthy controls**. To show comparisons of other diseases groups with healthy controls in the 1000 Genomes Project, we used several cancer types such as bladder cancer, breast cancer, colon cancer, kidney cancer, lung adenocarcinoma, lung squamous cell carcinoma, malignant melanoma, ovarian serous cystadenocarcinoma, prostate cancer and rectal cancer. The density plot of the rank is based on mutual information of the corresponding MeSH disease terms between each patient group and healthy controls (line colors: red, Patient group; black, African; orange, Asian; purple, American; cyan, European).

To compare the ranks of the disease terms in the AML patient group with those in the healthy control group, we used "leukemia, myeloid, acute" (C04.557.337.539.550) as the corresponding MeSH disease term (Figure 4). The red solid line in Figure 4 indicates the distribution of the rank of the disease term "leukemia, myeloid, acute" (C04.557.337.539.550) in the AML patient group, and the dashed lines indicate the distribution of the rank of the disease term in the healthy control group according to the various subpopulations. Notably, the AML patients have a substantially higher rank of this disease term than the healthy control group. In order to examine more other diseases than AML, we used several cancer types such as bladder cancer, breast cancer, colon cancer, kidney cancer, lung adenocarcinoma, lung squamous cell carcinoma, malignant melanoma, ovarian serous cystadenocarcinoma, prostate cancer and rectal cancer from TCGA and breast cancer and lung cancer from COSMIC. We generated density plot of the rank based on mutual information of the corresponding MeSH disease terms between each patient groups and healthy controls in the 1000 Genomes Project (Figure 5). The table in the Additional file 3 provides the MeSH codes of used sequencing data to compare rank based on mutual information between disease groups and healthy controls. The ranks of the corresponding disease MeSH codes in the all disease groups are higher than healthy controls. These results show that the methodology can be applied to infer associations between a personal genome and diseases.

### Evaluation of disease rank in the sequencing data

A quantitative comparison was made between the AML patient group and data for the healthy subpopulations in the 1000 Genomes Project in terms of the relative closeness between the genome and diseases based on the proposed method. We examined how highly the AML-related MeSH term "leukemia, myeloid, acute" (C04.557.337.539.550) was ranked in the two groups. Specifically, we treated the corresponding AML-related MeSH term as a true positive and computed the receiver operating characteristic (ROC) curve across the different thresholds. Overall, the area under the ROC curve of the AML patient data was significantly higher than that of the healthy control data from the 1000 Genomes Project, as indicated in Table 3. In order to explain evaluation more detail, we showed rank percentage between AML patient group and healthy subpopulations in the 1000 Genomes Project. The rank percentage is defined as the rank of "leukemia, myeloid, acute" (C04.557.337.539.550) as the corresponding MeSH disease term in the group such as AML patient group and healthy controls in the 1000 Genomes Project is divided by total number of MeSH term. Lower rank percentages mean higher rank of the corresponding MeSH term within the group. The bar plot of rank percentage shows that AML patient group have higher rank on "leukemia, myeloid, acute" (C04.557.337.539.550) than healthy controls (Additional file 4). These observations could highlight the importance of associating the genome sequencing data with the related diseases being used to interpret the personal genome with regard to diseases.

**Table 3:** Comparison of the area under ROC (AUC) between the AML patients and the healthy controls

	Patients	Healthy controls in the 1000 Genomes Project			
**Populations**	**AML**	**African**	**European**	**Asian**	**American**

AUC	0.992	0.676	0.680	0.683	0.687

## Discussion

This study had provided the first computational analysis scheme for interpreting the personal genome by simultaneously considering the functional impact of damaging variants and curated disease-gene associations. Herein we present a method for associating the personal genome with diseases based on the relative closeness between the personal genome and diseases.

The similarity structure patterns based on mutual information between the genomes and the diseases in the MeSH disease category differ according to the subpopulations in the 1000 Genomes Project data. One particularly notable finding was that the African subpopulations were clustered into one group, and the mutual information values were far higher for the African subpopulations than for the other subpopulations. We can connect this result with previous research in terms of the concept of the "recent African origin of modern humans." African populations are genetically more diverse than European and Asian populations [19-21]. According to the out-of-Africa hypothesis of human origins, this can be explained by groups migrating out of Africa experiencing severe population bottlenecks that have resulted in a reduction in the genetic diversity in the descendant populations [22,23]. A reduction in nucleotide diversity outside Africa has been consistently observed in genotype and resequencing data [22]. Due to the adaptive advantage of the heterozygote, sickle-cell anemia is prevalent, especially among people with recent ancestry in malaria-stricken areas, such as Africa [24]. The 1000 Genomes Project Consortium showed genetic variation within and between populations using the same data that this study used [25]. In the paper, they mentioned "individuals from populations with substantial African ancestry (YRI, LWK and ASW) carry up to three times as many low-frequency variants (0.5-5% frequency) as those of European or East Asian origin, reflecting ancestral bottlenecks in non-African populations". The reason that African population has higher overall mutual information in this study could ascribe to more number of low-frequency variants than other populations. This doesn't mean that the population is more sensitive to the disease but suggests that in order to interpret the personal genome properly, we should consider together population characteristics including genetic variations within populations.

Population-level disease-gene associations in this study are based on individual-level disease-gene associations. After getting each individual-level disease-gene associations, we summarized total individual-level disease-gene associations into population-level disease-gene associations. It is somewhat difficult to evaluate the proposed method and figure out biological or medical implications from individual observations. Characterized populations such as normal group or specific disease group can provide intuitive grasp in the purpose of evaluating the proposed method. After the proposed method is evaluated as proper method to interpret the genome sequencing data from population-level, we can apply this method to individual observations and interpret individual variations from personal genome data.

## Conclusions

In conclusion, this study could open up novel possibilities regarding the interpretation of personal genome sequencing. Rich disease-gene association knowledge can enable the accurate prediction of disease predisposition patterns. The scheme of this method could be expanded for applications in genomic-based knowledge, such as drug-gene and environmental-factor-gene interactions.

## Abbreviations

AML: acute myeloid leukemia; ANOVA: analysis of variance; ASW: African ancestry in southwest USA; COSMIC: Catalogue Of Somatic Mutations In Cancer; LWK: Luhya in Webuye, Kenya; MeSH: Medical Subject Headings; NGS: next-generation sequencing; OMIM: Online Mendelian Inheritance in Man; ROC: receiver operating characteristic; SIFT: Sorting Intolerant From Tolerant; TCGA: The Cancer Genome Atlas; Tukey's HSD test: Tukey's honest significant difference test; YRI: Yoruba in Ibadan, Nigeria

## Competing interests

The authors declare that they have no competing interests.

## Authors' contributions

JHK supervised YJN in bioinformatics and systems biology analyses. KAS helped performing the statistical data analyses. YJN conceived, designed, performed data analysis, and drafted the manuscript. All authors contributed to manuscript development. All of the authors had approved the final, submitted draft of the manuscript.

## Supplementary Material

Additional file 1 Comparison of the variability of mutual information with regard to MeSH disease categories in the 1000 Genomes Project data. (A) Bar plot of mutual information of statistically different MeSH categories in the 1000 Genomes Project data. Data are mean and SD values. The red horizontal line indicates the average mutual information of all populations in the 1000 Genomes Project. MI in the y-axis means mutual information. (B) Tukey's HSD test for post-ANOVA Comparisons of the MeSH categories: C15 - hemic and lymphatic diseases; C16 - congenital, hereditary, and neonatal diseases and abnormalities; and C17 - skin and connective-tissue diseases. Click here for file

Additional file 2 **Distribution of the rank in the 1000 Genomes Project data according to MeSH codes**. The density plots show the rank of each MeSH code in the healthy controls in the 1000 Genomes Project. The x-axis means ranks based on mutual information and the y-axis means kernel density. Click here for file

Additional file 3 **The MeSH code list of each disease**. The MeSH code list of each disease is used in order to compare ranks of MeSH disease terms in the disease groups (bladder cancer, breast cancer, colon cancer, kidney cancer, lung adenocarcinoma, lung squamous cell carcinoma, malignant melanoma, ovarian serous cystadenocarcinoma, prostate cancer and rectal cancer) with those in the healthy controls in the 1000 Genomes Project. Click here for file

Additional file 4 **Rank percentage between AML patient group and healthy subpopulations in the 1000 Genomes Project**. The rank percentage is defined as the rank of "leukemia, myeloid, acute" (C04.557.337.539.550) as the corresponding MeSH disease term in the group such as AML patient group and healthy controls in the 1000 Genomes Project is divided by total number of MeSH term. Click here for file

## Declarations

Publication of this article has been funded by the ICT R&D program of MSIP/IITP [B0101-15-247, Development of open ICT healing platform using personal health data]. This research was supported by the ICT R&D program of MSIP/IITP [B0101-15-247, Development of open ICT healing platform using personal health data] and by a grant of the Korean Health Technology R&D Project, Ministry of Health and Welfare (HI13C2164).

This article has been published as part of *BMC Medical Genomics *Volume 8 Supplement 2, 2015: Selected articles from the 4th Translational Bioinformatics Conference and the 8th International Conference on Systems Biology (TBC/ISB 2014). The full contents of the supplement are available online at http://www.biomedcentral.com/bmcmedgenomics/supplements/8/S2.
