# Supplementary figures and images for: Interpretation of personal genome sequencing data in terms of disease ranks based on mutual information
Source: BMC Med Genomics. 2015 May 29;8(Suppl 2):S4. doi: 10.1186/1755-8794-8-S2-S4 (PMC4460593; doi:10.1186/1755-8794-8-S2-S4)

(A)

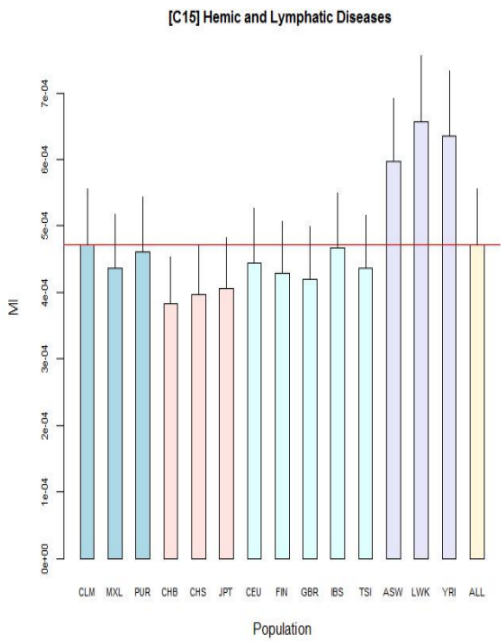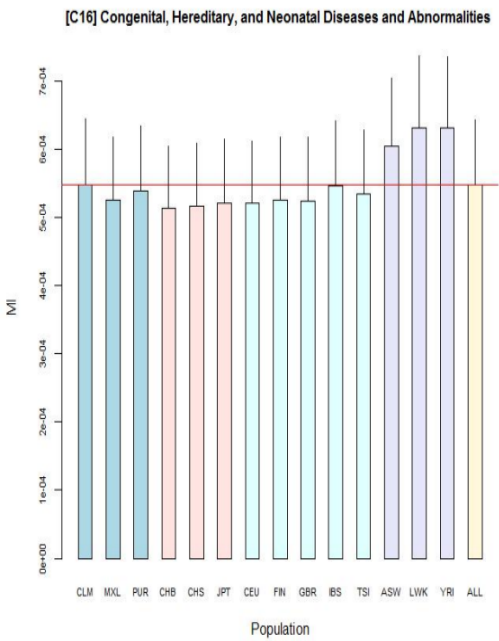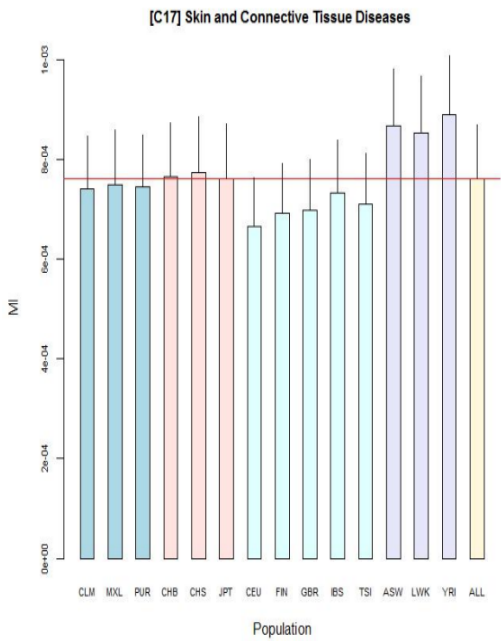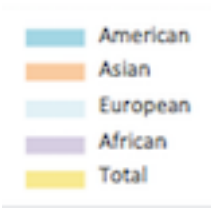

(B)

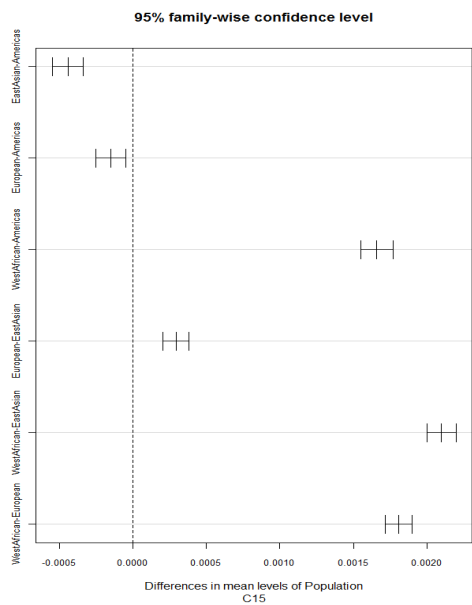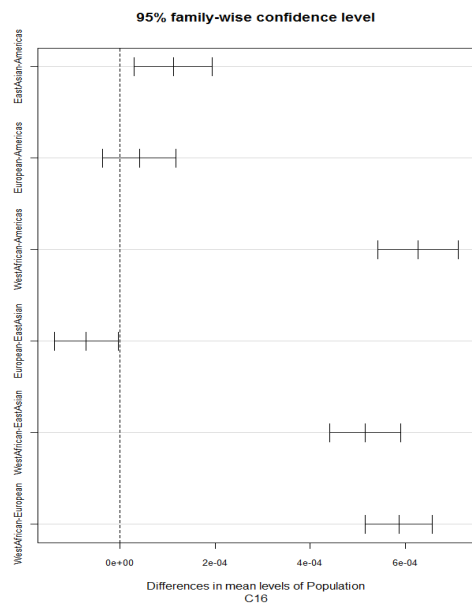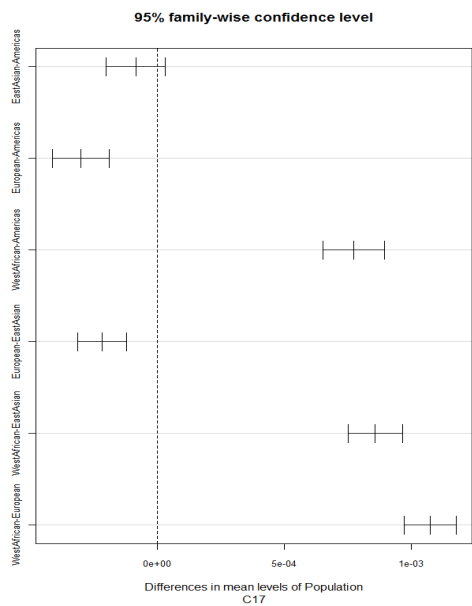

Supplement: Additional file 1 — Comparison of the variability of mutual information with regard to MeSH disease categories in the 1000 Genomes Project data. (A) Bar plot of mutual information of statistically different MeSH categories in the 1000 Genomes Project data. Data are mean and SD values. The red horizontal line indicates the average mutual information of all populations in the 1000 Genomes Project. MI in the y-axis means mutual information. (B) Tukey's HSD test for post-ANOVA Comparisons of the MeSH categories: C15 - hemic and lymphatic diseases; C16 - congenital, hereditary, and neonatal diseases and abnormalities; and C17 - skin and connective-tissue diseases. [file 1755-8794-8-S2-S4-S1.pdf]

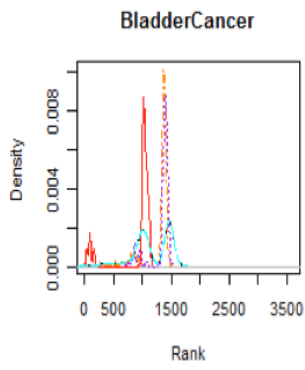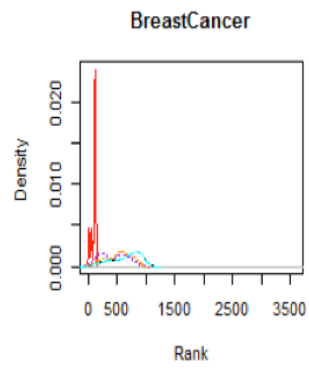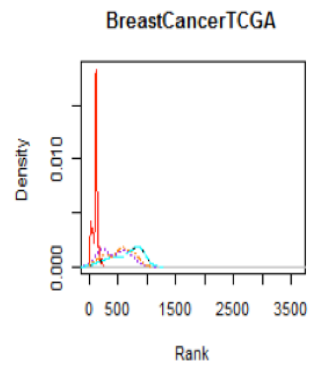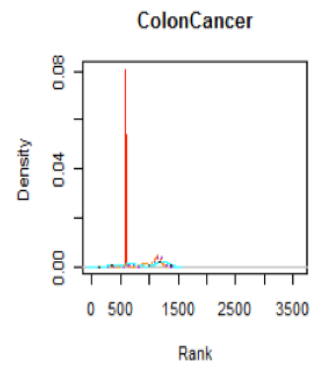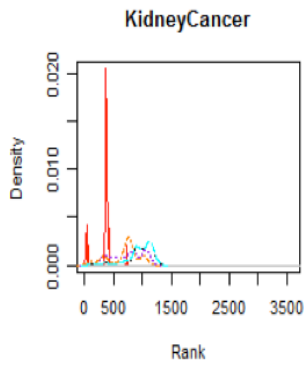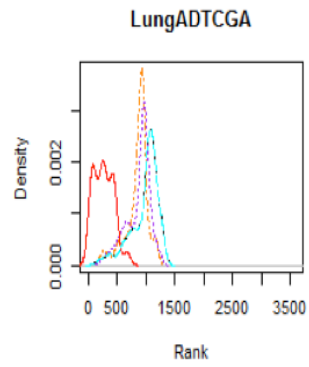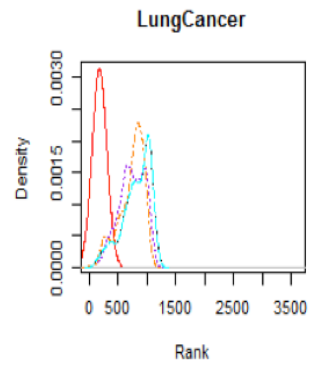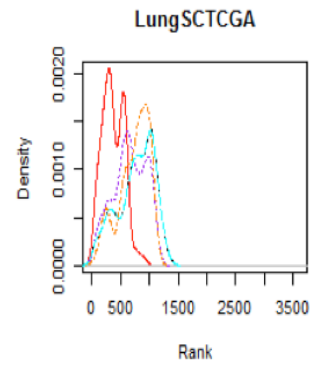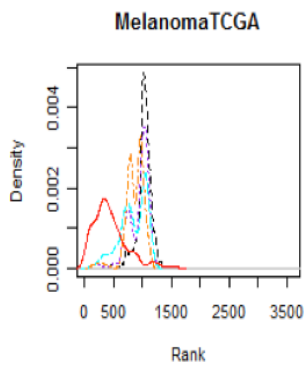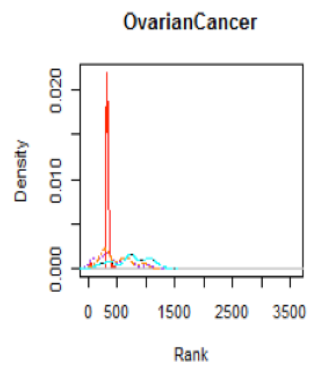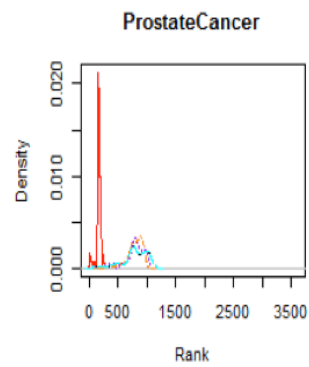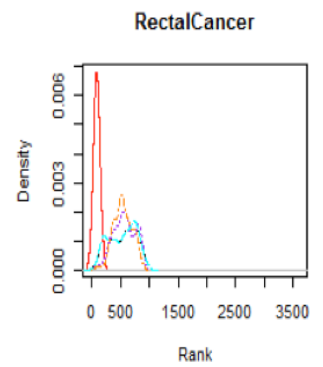

Supplement: Additional file 4 — Rank percentage between AML patient group and healthy subpopulations in the 1000 Genomes Project. The rank percentage is defined as the rank of "leukemia, myeloid, acute" (C04.557.337.539.550) as the corresponding MeSH disease term in the group such as AML patient group and healthy controls in the 1000 Genomes Project is divided by total number of MeSH term. [file 1755-8794-8-S2-S4-S4.pdf]
