# Supplementary material for: Interpretation of personal genome sequencing data in terms of disease ranks based on mutual information
Source: BMC Med Genomics. 2015 May 29;8(Suppl 2):S4. doi: 10.1186/1755-8794-8-S2-S4 (PMC4460593; doi:10.1186/1755-8794-8-S2-S4)
Supplement: Additional file 2 — Distribution of the rank in the 1000 Genomes Project data according to MeSH codes. The density plots show the rank of each MeSH code in the healthy controls in the 1000 Genomes Project. The x-axis means ranks based on mutual information and the y-axis means kernel density. [file 1755-8794-8-S2-S4-S2.pdf]

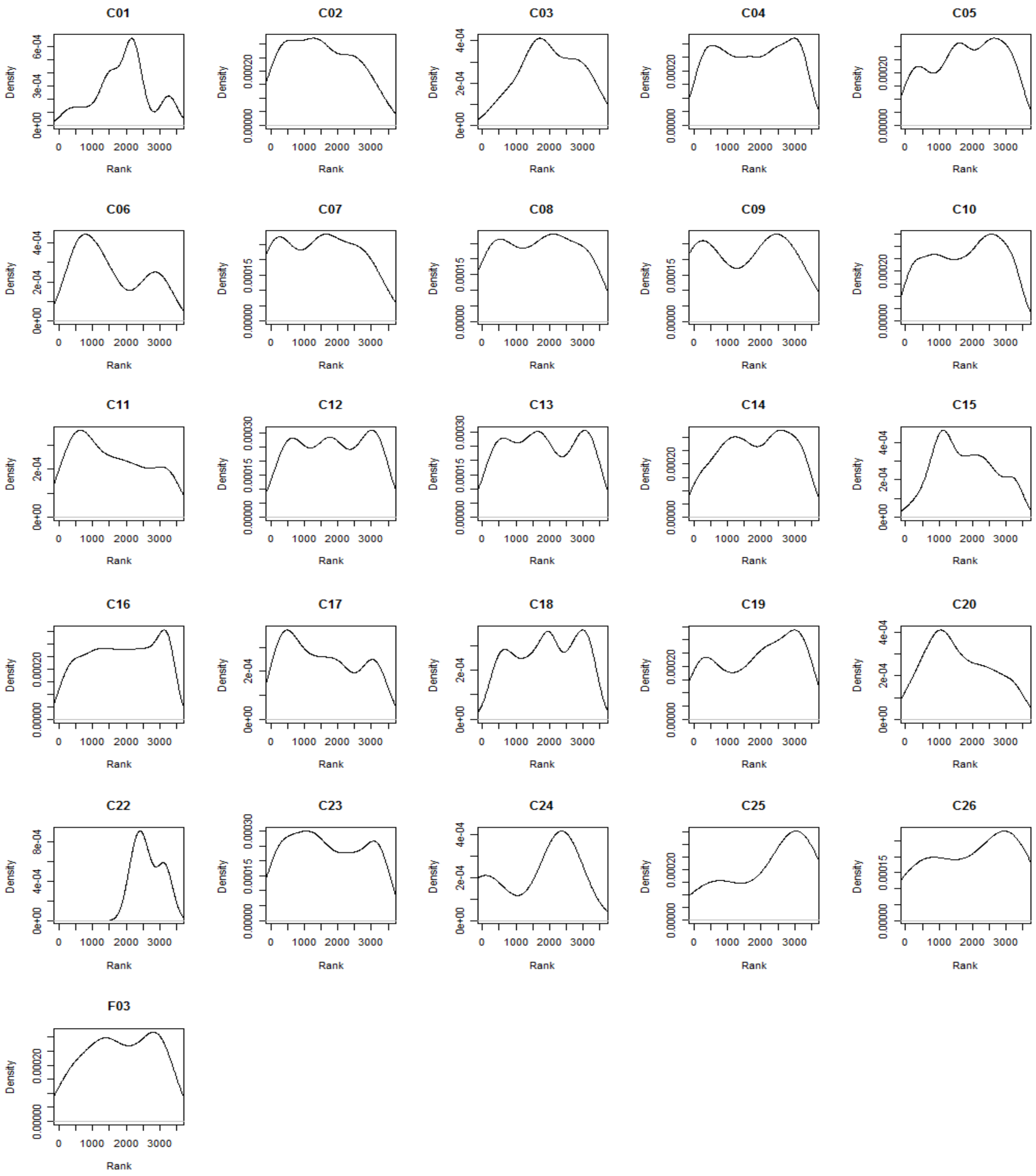
C01
C02
C03
C04
C05
C06
C07
C08
C09
C10
C11
C12
C13
C14
C15
C16
C17
C18
C19
C20
C22
C23
C24
C25
C26
F03
Density
Rank
0 1000 2000 3000

Code	Term Name
C01	Bacterial Infections and Mycoses
C02	Virus Diseases
C03	Parasitic Diseases
C04	Neoplasms
C05	Musculoskeletal Diseases
C06	Digestive System Diseases
C07	Stomatognathic Diseases
C08	Respiratory Tract Diseases
C09	Otorhinolaryngologic Diseases
C10	Nervous System Diseases
C11	Eye Diseases
C12	Male Urogenital Diseases
C13	Female Urogenital Diseases and Pregnancy Complications
C14	Cardiovascular Diseases
C15	Hemic and Lymphatic Diseases
C16	Congenital, Hereditary, and Neonatal Diseases and Abnormalities
C17	Skin and Connective Tissue Diseases
C18	Nutritional and Metabolic Diseases
C19	Endocrine System Diseases
C20	Immune System Diseases
C21	Disorders of Environmental Origin
C22	Animal Diseases
C23	Pathological Conditions, Signs and Symptoms
C24	Occupational Diseases
C25	Substance-Related Disorders
C26	Wounds and Injuries
F03	Mental Disorders
