# Supplementary material for: Interpretation of personal genome sequencing data in terms of disease ranks based on mutual information
Source: BMC Med Genomics. 2015 May 29;8(Suppl 2):S4. doi: 10.1186/1755-8794-8-S2-S4 (PMC4460593; doi:10.1186/1755-8794-8-S2-S4)
Supplement: Additional file 3 — The MeSH code list of each disease. The MeSH code list of each disease is used in order to compare ranks of MeSH disease terms in the disease groups (bladder cancer, breast cancer, colon cancer, kidney cancer, lung adenocarcinoma, lung squamous cell carcinoma, malignant melanoma, ovarian serous cystadenocarcinoma, prostate cancer and rectal cancer) with those in the healthy controls in the 1000 Genomes Project. [file 1755-8794-8-S2-S4-S3.pdf]

Disease	MeSH Disease terms	No. of MeSH codes	MeSH code
Acute Myeloid Leukemia	Leukemia, Myeloid, Acute	1	C04.557.337.539.550
Bladder Urothelial Carcinoma	Urinary Bladder Neoplasms	5	C04.588.945.947.960, C12.758.820.968, C12.777.829.813, C13.351.937.820.945, C13.351.968.829.707
Breast Carcinoma	Breast Neoplasms	2	C04.588.180, C17.800.090.500
Colon adenocarcinoma	Colonic Neoplasms	5	C04.588.274.476.411.307.180, C06.301.371.411.307.180, C06.405.249.411.307.180, C06.405.469.158.356.180, C06.405.469.491.307.180
Kidney renal clear cell carcinoma	Carcinoma, Renal Cell	6	C04.557.470.200.025.390, C04.588.945.947.535.160, C12.758.820.750.160, C12.777.419.473.160, C13.351.937.820.535.160, C13.351.968.419.473.160
Small Cell Lung Carcinoma	Lung Neoplasms<br>Small Cell Lung Carcinoma	6	C04.588.894.797.520, C08.381.540, C08.785.520, C04.588.894.797.520.109.220.624, C08.381.540.140.750, C08.785.520.100.220.750
Lung adenocarcinoma	Lung Neoplasms	3	C04.588.894.797.520, C08.381.540, C08.785.520
Lung squamous cell carcinoma	Lung Neoplasms<br>Carcinoma, Squamous Cell	5	C04.588.894.797.520, C08.381.540, C08.785.520, C04.557.470.200.400, C04.557.470.700.400
Melanoma	Melanoma	3	C04.557.465.625.650.510, C04.557.580.625.650.510, C04.557.665.510
Ovarian Neoplasms	Ovarian Neoplasms	5	C04.588.322.455, C13.351.500.056.630.705, C13.351.937.418.685, C19.344.410, C19.391.630.705
Prostate adenocarcinoma	Prostatic Neoplasms	4	C04.588.945.440.770, C12.294.260.750, C12.294.565.625, C12.758.409.750
Rectum adenocarcinoma	Rectal Neoplasms	5	C04.588.274.476.411.307.790, C06.301.371.411.307.790, C06.405.249.411.307.790, C06.405.469.491.307.790, C06.405.469.860.180.500
